# Plaque Erosion: A Distinctive Pathological Mechanism of Acute Coronary Syndrome

**DOI:** 10.3389/fcvm.2021.711453

**Published:** 2021-09-28

**Authors:** Xing Luo, Ying Lv, Xiaoxuan Bai, Jinyu Qi, Xiuzhu Weng, Shaoyu Liu, Xiaoyi Bao, Haibo Jia, Bo Yu

**Affiliations:** ^1^Department of Cardiology, 2nd Affiliated Hospital of Harbin Medical University, Harbin, China; ^2^Key Laboratory of Myocardial Ischemia, Ministry of Education, Harbin Medical University, Harbin, China; ^3^Bin Xian People's Hospital, Harbin, China

**Keywords:** acute coronary syndrome, plaque erosion, plaque rupture, optical coherence tomography, biomarker 2

## Abstract

Plaque erosion (PE) is one of the most important pathological mechanisms underlying acute coronary syndrome (ACS). The incidence of PE is being increasingly recognized owing to the development and popularization of intracavitary imaging. Unlike traditional vulnerable plaques, eroded plaques have unique pathological characteristics. Moreover, recent studies have revealed that there are differences in the physiopathological mechanisms, biomarkers, and clinical outcomes between PE and plaque rupture (PR). Accurate diagnosis and treatment of eroded plaques require an understanding of the pathogenesis of PE. In this review, we summarize recent scientific discoveries of the pathological characteristics, mechanisms, biomarkers, clinical strategies, and prognosis in patients with PE.

## Introduction

Acute coronary syndrome (ACS) is a common and highly fatal cardiovascular disease. It has been considered that the rupture of atherosclerotic vulnerable plaques is the main pathological mechanism of ACS (1). Some progress has been made in the understanding of the pathogenesis and development of preventive measures against so-called “vulnerable plaques” (2, 3). Through the application of intraluminal imaging, especially optical coherence tomography (OCT), it was found that plaque erosion (PE) is also an important mechanism underlying ACS (4). There is increasing evidence that patients with eroded plaques are different from patients with traditional ruptured plaques in the physiopathological mechanisms, predictive biomarkers, and prognosis (5, 6). Thus, understanding the pathologic characteristics and pathogenesis of PE may be helpful for the development of individualized treatment strategies for patients with eroded plaques.

## Pathological Features of Eroded Plaques

**Figure 1:**
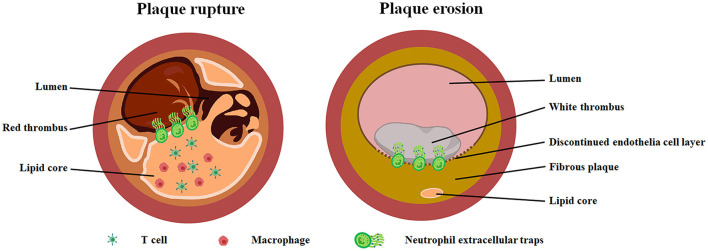
Pathological characteristics of plaque rupture and plaque erosion. Ruptured plaque (left image) is featured with a larger lipid core containing abundant macrophages and T cells. Red thrombus was observed in the small lumen. Eroded plaque (right image) has a large lumen with white thrombus and fibrous plaque tissue characterized by little or no lipid deposition. In particularly, there is discontinuous endothelial cell layer in eroded plaque. Neutrophil extracellular traps was found at the junction of plaque tissue and thrombus in both eroded and ruptured plaque.

There is evidence indicating that it is not only the rupture of the fibrous cap of lipid plaques that could lead to acute thrombosis and sudden cardiac death. According to recent studies (1, 4, 7, 8), approximately one-third of the cases of ST-segment elevation myocardial infarction (STEMI) did not present with PR, but rather, exhibited PE. Autopsy and imaging studies indicate that PE manifests different pathological features from those of PR (Figure 1). It has been reported that PR is characterized by a thin fibrous cap and discontinuous intimal layer. The local endothelial cells exist, but are dysfunctional in ruptured plaque, whereas PE exhibits an intact and thick fibrous cap, and the local endothelial cells are missing (7). The disturbance of lipid metabolism is the main feature of patients with PR (9). A larger necrotic core, including accumulated lipids and inflammatory cells, is often observed in PR. In contrast, PE is recognized as fibrous plaques rich in smooth muscle cell and extracellular matrix, such as hyaluronic acid (HA) and proteoglycan, but with fewer macrophages and T cells. The lipid core is also small or not visible. Compared to ruptured plaques, eroded plaques possess a large lumen area, and it was found that more than half of the eroded plaques are presented with <75% area stenosis (10). Nevertheless, not all eroded plaques are the same, Dai et al. divided PE into three subtypes based on the plaque morphology characterized by OCT: eroded plaque (fibrous plaque), defined as a lesion with high backscattering and a homogeneous region; eroded plaque (thick-cap fibroatheroma, ThcFA), defined as a lesion with minimal fibrous cap thickness ≥65 μm; and eroded plaque (thin-cap fibroatheroma, TCFA), defined as a lesion with minimal fibrous cap thickness <65 μm, account for 50, 36.5, and 13.5% of PE, respectively (11). Moreover, the composition of the thrombus formed by PE is also different from that of PR. In ruptured plaques, red thrombi mainly composed of fibrin and red blood cells are formed, whereas the surface of the eroded plaque was mainly white thrombus dominated by platelets and fibrinogen (12). OCT was used to analyze the contents of the residual thrombus after successful reperfusion with fibrinolysis in PE and PR; the results indicated that the white thrombus was the predominant type at the culprit site, whereas the red thrombus was observed in the entire culprit lesion of PR patients (13).

## Potential Pathophysiology of PE

**Figure 2:**
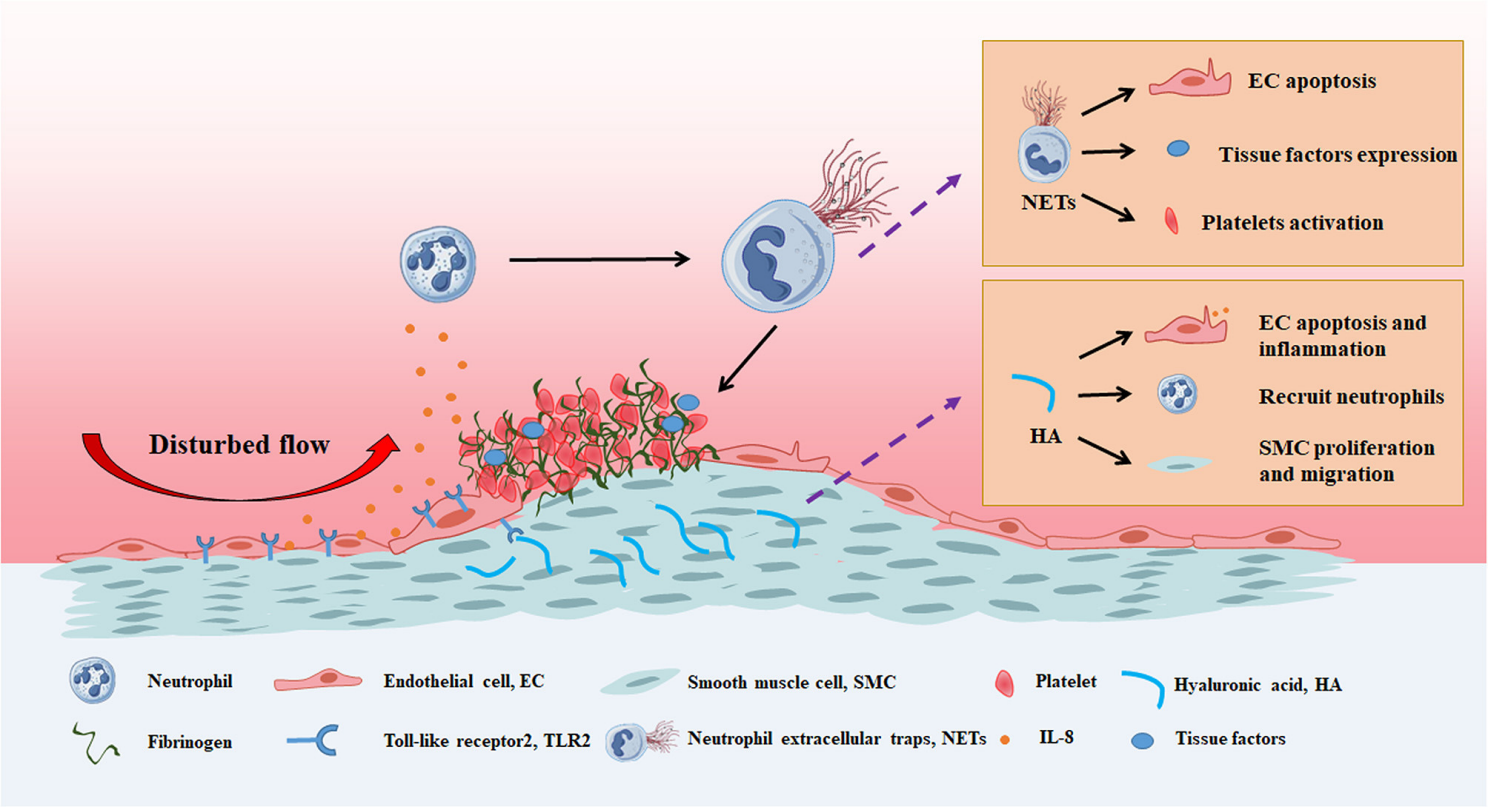
Possible mechanisms between disturbed flow, neutrophil extracellular traps and hyaluronic acid in eroded plaque. The disturbed flow triggers the TLR2 dependent endothelial cell apoptosis and secretion of IL-8, leading to neutrophil recruitment, activation and release of neutrophil extracellular traps, aggravate the injury of endothelial cell layer and enhances the thrombus formation. In addition, accumulated hyaluronic acid induces apoptosis and inflammation in endothelial cells, followed by neutrophils adhesion. Hyaluronic acid also promotes the proliferation and migration of smooth muscle cells.

With the increase in number of pathologic and imaging studies on PE, the mechanisms of PE have drawn much attention. There are several emerging concepts regarding the underlying mechanism of PE (Figure 2).

HA, an extracellular matrix (ECM) polysaccharide with different molecular weights, is generally synthesized by multiple cell types in the vascular wall. Endogenous HA can be degraded faster by hyaluronidases (HYALs) and reactive oxygen species (ROS) under pathological conditions (14). The accumulation and disturbance of HA at the border of the plaque/thrombus interface has been proven to be an important pathological feature of eroded plaque (15, 16). In addition, it has been reported that there are elevated levels of HYAL2 and CD44v6 (a variant of the hyaluronan binding protein) in peripheral blood mononuclear cells isolated from patients with eroded plaques compared to patients with stable angina or without coronary disease, which supports a central role for hyaluronan in eroded plaques (17). Furthermore, evidence was presented in an erosion-like animal model in which enzymes involved in the synthesis and turnover of hyaluronan in this mouse model were altered, showing decreased hyaluronic acid synthase2 (HAS2) and HYAL1 mRNA levels; the accumulation of HA was also as in eroded lesions (18). Mechanically, HA was shown to bind to a variety of receptors that regulate cellular functions, such as CD44 and HA-mediated motility receptor. Recent studies demonstrated that HA could promote smooth muscle cell proliferation and migration, raise inflammatory cells, and trigger the coagulation cascade (19–23). Moreover, studies focused on the effect of HA on EC function revealed that HA promoted EC stress and apoptosis, aggravated endothelial activation, and impaired EC adherence via the toll-like receptor 2 (TLR2) dependent pathway (19). These *in vitro* studies indicated that HA may accelerate the progression of eroded plaques. In particular, Libby hypothesized that low molecular weight HA promotes IL-8 production in endothelial cells via the NF-κB pathway, which triggers Neutrophil extracellular traps (NETs) formation and the activation of platelets (18). Generally, metabolic disorder of HA was proven to be the core event in the onset of PE, which is closely related to EC apoptosis, the type conversions of SMC, and neutrophil activation.

NETs, including cell-free DNA, myeloperoxidase (MPO), and neutrophil elastase (NE), are fibrous networks that protrude from the membranes of activated neutrophils (24). Several autopsy studies revealed that NETs exist on the surface of culprit lesions in patients with PE (19, 25–27). In addition, Yan and Ferrante reported that patients with PE exhibited higher MPO plasma levels than PR (28, 29). The formation of NETs requires peptidyl arginine deiminase (PAD) 4. Indeed, bone marrow deficiency of PAD4 reduced NETs formation in atherosclerosis, but did not alter the number of circulating neutrophils and the ability to adhere to endothelial cells. It is surprising that LdLr-/- mice with PAD4-/- bone marrow showed no non-significant effect on the progression of atherosclerosis. However, mice PAD4-/- bone marrow abolished the growth of NETs and prevented the damage of endothelial cells and thrombus formation. The same effect was also observed in DNaseI (a type of enzyme for degrading NETs) administration in mice (27). The role of NETs in PE is thought to involve damage to the EC layers and enhancement of thrombus formation. Libby found that MPO can locally generate hypochlorous acid, a potent pro-oxidant that can induce endothelial cell apoptosis and tissue factor expression (30). NE and cell-free DNA also showed significant cytotoxicity in endothelial cells (31, 32). On the other hand, it has been previously reported that cell-free DNA binds circulating platelets and coagulation factors, acting as a solid-state reactor for the coagulation cascade (33–35). In addition, MPO and NE were proven to induce EC activation and tissue factor production (36–39). Collectively, these studies indicate that the elevated levels of NETs observed on the surface of culprit lesions may contribute causally to endothelial cell injury and coronary thrombosis formation in eroded plaques.

Accumulating evidence suggests that a disturbed flow contributes to altered endothelial function, which can predispose to PE (18). Clinical research has indicated that PE is more likely to occur in vessel bifurcation, which is characteristic of disturbed flow (9, 40–42). Several computational fluid dynamics combined OCT studies reported that a change of local hemodynamics accompanied with high endothelial shear stress (ESS) was observed in the critical lesion of PE patients (41, 43, 44). Furthermore, Jang and collogues compared ESS, spatial ESS gradient (ESSG), and oscillatory shear index (OSI) between eroded plaques and ruptured plaques, the results shown that ESSG is higher at rupture sites than erosion sites, OSI is higher at erosion sites and ESS was similar (45). Moreover, the effect of high ESS on eroded plaques was verified *in vivo*. A rabbit model of SMC-rich atherosclerotic plaque was established to investigate the relationship between the magnitude of ESS and erosive injury, and the results demonstrated that high ESS was significantly correlated with the extent of histologically defined PE (46). In addition, Asada et al. created an SMC-rich plaque by balloon injury, and a vascular occlude was used to disturb blood flow after 3 weeks. They reported that EC detachment, platelet adhesion, and neointimal cell apoptosis became evident in the post-stenotic regions of all femoral arteries within 15 min of narrowing, followed by mural thrombi formation in 60 min, suggesting that oscillatory shear stress contributes to EC detachment and thrombus formation in PE (47). Furthermore, the pro-erosion effect of disturbed flow was also observed in mice, and Libby et al. performed a superficial erosion mouse model by combining shock injuries and blood flow disorders with a vascular occlude (18). Taken together, these data demonstrate that disturbed flow plays a vital role in the progression of eroded plaques.

Recent studies have focused on the mechanisms by which disturbed flow promotes the progression of PE. TLR2, an innate immune receptor, was observed to show higher expression in PE critical lesions than in stable plaques and ruptured plaques. Moreover, TLR2 staining in the eroded plaque was co-localized with EC and neutrophils (19). To study the relationship between disturbed flow and TLR2, an erosion-like mouse model from Libby's group was established via intimal injury combined with vascular narrowing. After these procedures, the pathological characteristics of eroded plaque, including EC detachment, HA accumulation, platelet adhesion, neutrophil infiltration, and SMC-rich neointimal, were clearly displayed. However, mice deficient in TLR2 showed resistance to these procedures, which suggested that disturbed flow, contributed to the onset of PE and is dependent on TLR2 activation (18). An *in vitro* study indicated that TLR2 agonists, including HA, promote EC dysfunction by increasing the expression of chemokines, endoplasmic reticulum stress, excessive ROS production, and apoptosis (18, 19). This observation further supported the hypothesis that disturbed flow leads to the endogenous activation of TLR2 in EC; chemokines, including IL-8, are secreted to promote neutrophil recruitment and induce NET formation in eroded plaques.

## Biomarkers and Prediction Tools in Patients With PE

**Figure 3:**
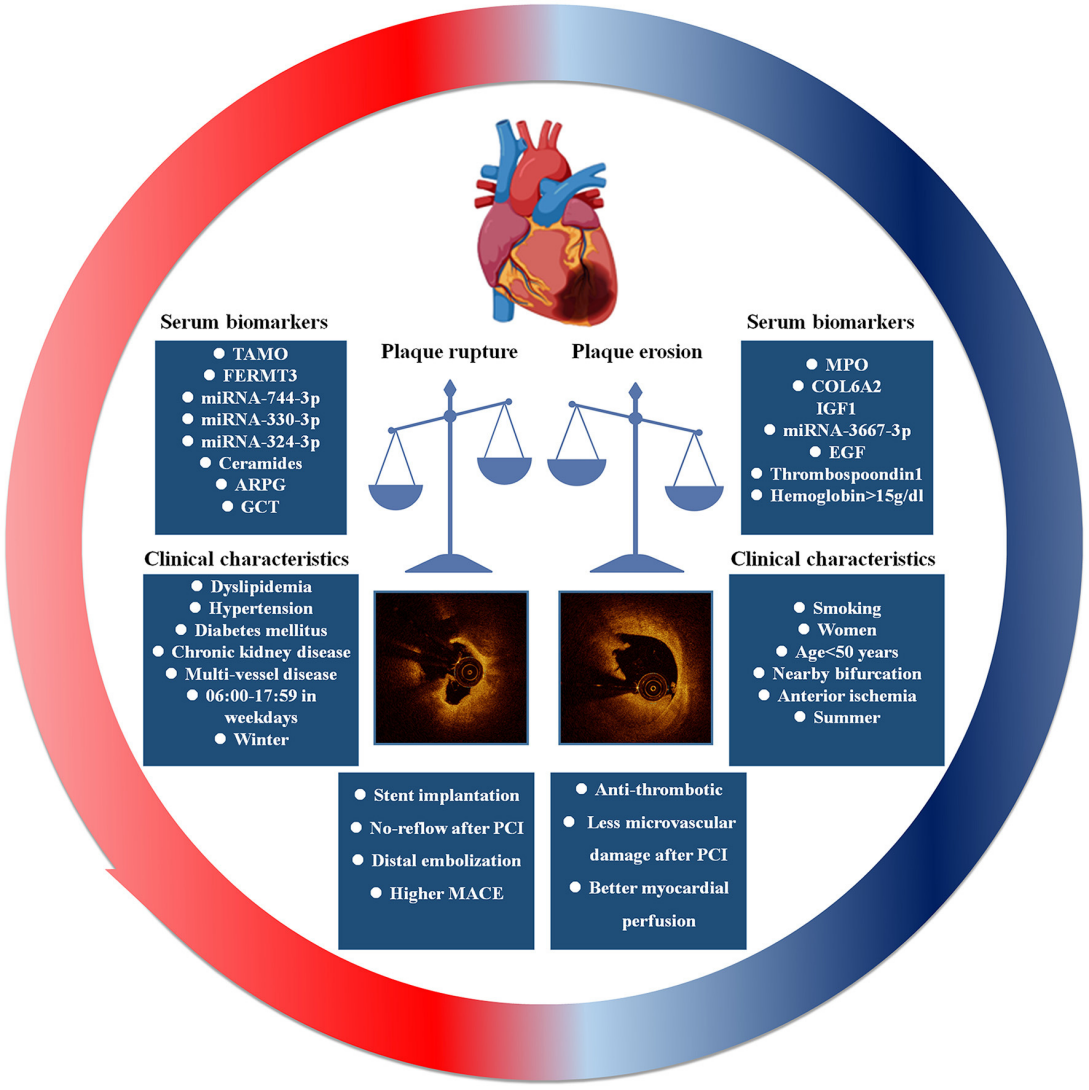
The prediction and clinical outcome of plaque erosion and plaque rupture.

To date, OCT is the only intracavity imaging method that can accurately diagnose PE *in vivo*. In order to diagnose PE quickly and non-invasively, biomarkers for predicting PE and PR have been well-researched (Figure 3). An autopsy study reported that the density of MPO-positive cells within thrombi overlying plaques was significantly higher in lesions with erosion than in ruptures, this study concomitantly compared the systemic MPO level and C-reactive protein (CRP) in 25 patients with ACS and showed that the systemic levels of serum MPO were significantly higher in patients with eroded plaques than in those with ruptured plaques, but there were no differences in the level of CRP between the two groups (28). However, the results of another autopsy study were not in agreement with those results, they reported that NETs, including MPO, were abundantly present in eroded and ruptured plaques, and MPO was observed in the thrombus and at the thrombus–plaque interface, with no significant difference in expression between the eroded and ruptured plaque (26). To investigate the association between plasma MPO levels and PE, a prospective study that enrolled 172 STEMI patients diagnosed by OCT was conducted. This study revealed that, compared to PR, the plasma level of MPO was significantly higher in patients with PE, and multivariable logistic regression showed that plasma MPO was independently associated with PE (odds ratio: 3.25; 95% confidence interval: 1.37–7.76; *p* = 0.008). However, the results from the receiver-operating characteristic curve (ROC) were not satisfied, the area under the ROC was 0.75, which suggested that MPO had moderate diagnostic ability for PE; in order to improve diagnostic accuracy, other indicators must be combined (29).

In recent years, omics have been widely used to discover disease biomarkers and predict disease outcomes. Liu and collogues conducted a high-throughput tandem mass tag (TMT)-based comparative proteomics to identify PR-and PE-specific biomarkers; 36 significant differential proteins were identified among groups, after validation with enzyme-linked immunosorbent assay; the results showed that elevated levels of collagen type VI α-2 chain (COL6A2) and insulin-like growth factor 1 (IGF1), and decreased levels of fermitin family homolog 3 (FERMT3) were positively associated with patients with PE. Moreover, multivariate analysis indicated that the plasma levels of IGF1, FERMT3, and COL6A2 could predict PE independently (48). In addition, another miRNA microarray study from our group also reported that high levels of miRNA-3667-3p in plasma are associated with PE in patients with STEMI, and expanded the number of patients verified results showing that miRNA-3667-3p exhibited a significant discriminatory power in predicting the presence of PE (AUC = 0.767) (49). Subsequently, another miRNA sequencing was performed to explore the biomarkers of PE, and the results demonstrated that miR-744-3p, miR-330-3p, and miR-324-3p in serum could be potential biomarkers to predict the plaque phenotype in STEMI patients (50). In addition, much attention has been focused on the relationship between intestinal flora homeostasis and the development of atherosclerotic plaques (51–53). Trimethylamine N-oxide (TMAO), a gut microbiota metabolite derived from dietary phosphatidylcholine and choline, has been shown to promote the progression of atherosclerosis and is associated with the risk of cardiovascular disease (54–57). To investigate this, the Yan' team prospectively enrolled 211 STEMI patients and detected the serum TMAO level by using stable isotope dilution liquid chromatography tandem mass spectrometry. The results indicated that the level of TMAO in serum was significantly higher in patients with ruptured plaque than in patients with eroded plaque. ROC analysis revealed the area under the ROC curve was 0.89 with a power of prediction, when the cutoff value was 1.95 μM; TMAO had a sensitivity of 88.3% and specificity of 76.8% to predict PR (58). Ceramides, sphingolipid product of sphingomyelinase, have been shown to trigger various apoptosis-and inflammation-related signaling pathways and the development of plaque progression (59–61). Another targeted metabolomics research from our group suggested that evaluated plasma ceramides is an independent predictor of PR in STEMI patients (62). Several studies have claimed that ruptured plaques show different inflammation levels to eroded plaques (63–65). Cytokine arrays were used to analyze the differential expression of 102 cytokines from the infarct-related artery and peripheral artery, and the different cytokines were confirmed in blood samples and thrombectomy samples. The results demonstrated that the plasma levels of epidermal growth factor (EGF) and thrombospondin 1 were higher in patients with PE, and the mRNA of EGF was more highly expressed in eroded plaque thrombectomy on the surface of eroded plaque, but there were no differences in the expression of thrombospondin 1 (66).

In addition to plasma biomarkers, clinical characteristics and laboratory tests have also been proven to predict PE. Dai et al. prospectively enrolled 822 STEMI patients who underwent pre-intervention OCT, compared the characteristics of clinical, angiographic, and OCT findings and demonstrated that age <50 years, current smoking, absence of other coronary risk factors, lack of multi-vessel disease, reduced lesion severity, larger vessel size, and nearby bifurcation were significantly associated with PE. Nearby bifurcation and current smoking were especially notable in men, while age <50 years was the most predictive in women (9). Comparing to PR, PE was reported to prefer clustering near the bifurcation (PE 59.3 vs. PR 34.6%), particularly in LAD, the bifurcation was an independent anatomic predictor of PE (9). Another study divides patients with OCT-erosion into three groups according to severity of lumen area stenosis: area stenosis <50%, area stenosis = 50–75% and area stenosis >75%, they found that nearby bifurcation was independently associated with OCT-erosion with non-critical stenosis (<75% lumen area stenosis; vs. OCT-erosion with ≥75% area stenosis) (10). To further understand the local hemodynamics in eroded and ruptured plaque, three-dimensional reconstruction and computational fluid dynamics were performed to analysis ESS, spatial ESS gradient (ESSG) and oscillatory shear index (OSI) between plaque rupture and erosion through mixed-effects logistic regression, these results demonstrated that high ESSG is independently associated with PR while high ESSG, ESS, and OSI associate with PE (45). Moreover, patients with PE were more frequently current smokers (PE 63.6 vs. PR 52.1%), and current smoker is significantly associated with PE in the multivariable analysis (odds ratio = 1.56, *p* < 0.05). However, the association between current smoking and PE was not observed in women (odds ratio = 1.25, *p* = 0.52) but in men (odds ratio = 1.67, *p* < 0.05). These results indicate that PE might be a predictable clinical entity that is different from that of PR (9). Similar to clinical characteristics, laboratory tests have also been proven to predict PE. Jang et al. reported that age <68 years, anterior ischemia, absence of diabetes mellitus, hemoglobin >15.0 g/dL, and normal renal function were the five independent parameters associated with PE. When a non-STEMI patient had all five parameters, the probability of plaque erosion increased to 73.1% (5). According to Jang's findings, a history of diabetes mellitus was a predictor of plaque rupture in STEMI patients, but the predictive ability of glucose-related variables is not clear. Liu et al. studied the relationship between glucose-related variables and PR risk in STEMI patients [glucose-related variables included random plasma glucose on admission (ARPG), glycosylated hemoglobin (HbA1c), post-PCI fasting plasma glucose, acute-to-chronic glycemic ratio (A/C), glucose variable tendency (GVT), and DM history]. The results confirmed that ARPG and GVT are independent predictors for PR in non-diabetic patients; the higher the ARPG level, the greater the risk of PR in STEMI patients (67).

Interestingly, circadian and seasonal variations are associated with the pathological mechanism of ACS. Jang reported that PR is more likely to occur during winter, whereas PE is more likely to occur in summer (68). The authors thought that systemic inflammation induced by respiratory infections and increased blood pressure in winter contributed to plaque destabilization. In summer, the hot environment leads to the evaluated local endothelial shear stress by increasing the blood viscosity. In another study, Jang claimed that the incidence of PR peaks at 9:00, compared to the period of 00:00–05:59, the risk of PR was increased in the periods of 06:00–11:59 and 12:00–17:59, but this phenomenon only appeared during weekdays, but not during weekends. Circadian variations were not observed in PE or calcified plaques (69).

## Clinical Strategies and Outcomes in Patients With PE

The introduction of OCT enables us to distinguish PE *in vivo*, which allows cardiologists to evaluate the clinical treatment strategies and outcomes in patients with PE. Current standard of care mandates immediate stenting for STEMI, and usually an early invasive strategy with stenting for many cases of non-STEMI, which is called “one-size-fits-all” clinical strategy (70). However, considering the distinct demographic characteristics and pathologies of PE and PR, individualized treatment strategies are of vital importance.

A comparison of the angiographic characteristics of patients with PE and PR revealed that patients with PE had fewer multivessel lesions, lower Gensini risk scores, more preserved vascular structure and a larger lumen (71). All of these findings suggests that PE may not necessarily require stent implantation (Table 1). A single-center, uncontrolled, prospective, proof-of-concept study was performed to evaluate the feasibility and safety of long-term antithrombotic therapy without stenting in patients with ACS caused by PE. Patients with residual diameter stenosis <70% on coronary angiography and diagnosed with PE by OCT were treated with anti-thrombotic therapy without stent implantation (72). Fifty-five patients completed the 1-month follow-up period. One patient died of gastrointestinal bleeding, and another required repeat percutaneous coronary intervention (PCI). The remaining patients were asymptomatic. Thrombus volume decreased from 3.7 (range: 1.3–10.9) mm^3^ to 0.2 (range: 0.0–2.0) mm^3^ (72). At 1-year follow-up, OCT demonstrated that the median residual thrombus volume decreased significantly from 1 month to 1 year (0.3 mm^3^ vs. 0.1 mm^3^) (79). The majority 92.5% of patients with ACS caused by PE managed with antithrombotic therapy without stent implantation remained free of major adverse cardiovascular events for up to 1 year (79). Sugiyama et al. evaluated whether tirofiban, a glycoprotein IIb/IIIa inhibitor, had additional benefits in ACS patients with PE treated with antiplatelet therapy without stent implantation. The tirofiban group had smaller residual thrombus volume, thrombus burden, and greater reduction of thrombus volume at the 1-month follow-up. These differences were maintained for up to 1 year. There was no difference in the 1-year major adverse cardiovascular event rate between the two groups (80). Above all, the results suggested that anti-thrombotic instead of stent implantation maybe safe and effective for partial patients with PE.

**Table 1:** Representative studies of the clinical strategies and prognosis of the patients caused by plaque erosion.

**References**	**Number in study**	**Strategy**	**Follow up time**	**End point**	**Key observations**
Jia et al. (72)	55 patients with ACS caused by PE	Residual diameter stenosis <70% on coronary angiogram were treated with anti-thrombotic therapy without stenting.	1 month	The primary endpoint was >50% reduction of thrombus volume at 1 month compared with baseline. The secondary endpoint was a composite of cardiac death, recurrent ischaemiarequiring revascularization, stroke, and major bleeding.	47 patients met the primary endpoint, and 22 patients had no visible thrombus at 1 month.
Prati et al. (73)	31 patinents with STEMI	40% of patients with subcritically occlusive plaque were treated with dual antiplatelet therapy without PCI, and the remaining 60% of patients underwent angioplasty and stenting.	753 days	Myocardial infarction, heart failure, or deaths.	All patients were asymptomatic, regardless of stent implantation.
Souteyrand et al. (74)	46 patients with ACS, 39.1% of PR and 54.3% of PE.	Medical therapy treatment alone without stenting in case of absence of vulnerable plaque rupture and < 70% stenosis.	12 months	MACE	23 patients benefited from systematic delayed OCT over a median period of 171 days, showing an increase in minimal lumen area.
Hu et al. (75)	141 patients with ACS, 79 of PR and 62 of PE	Stent implantation was performed in 77 (97.5%) patients with PR versus 49 (79.0%) in those with PE.	12 months	MACE	OCT showed a higher incidence of malapposition (37.5% versus 7.3%, P <0.001), thrombus (59.4% versus 14.6%, P <0.001), and protrusion (93.8% versus 73.2%, P=0.008) in the rupture group compared with the erosion group.
Saia et al. (76)	32 plaques (33.0%) with an IFC, 63 (64.9%) plaques with a RFC.	Primary PCI with everolimus-eluting stent implatation	2 years	MACE	At the 9-month OCT, IFC and RFC had similar high rates of stent strut coverage (92.5% vs. 91.2%; p $\frac{1}{4}$ 0.15) and similar percentage of volume obstruction (12.6% vs. 10.2%; p $\frac{1}{4}$ 0.27). No significant differences in clinical outcomes were observed up to 2 years.
Higuma et al. (77)	112 patients with STEMI, 64.3% of PR and 26.8 of PE.	Primary PCI	No follow-up visits	–	PE had a lower incidence of no-reflow phenomenon after PCI than PR and associated with less microvascular damage after PCI.
Niccoli et al. (78)	139 patients with ACS, 82 of PR and 57 of IFC.	Primary PCI	31.58+4.69 months	MACE	Major adverse cardiac events occurred more frequently in patients with PR when compared with those having IFC (39.0 vs. 14.0%, P $\frac{1}{4}$ 0.001).

*ACS, acute coronary syndrome; STEMI, ST-segment elevation myocardial infarction; PCI, percutaneous coronary intervention; PE, plaque erosion; PR, plaque rupture; MACE, major adverse cardiac events; OCT, optical coherence tomography; IFC, intact fibrous cap; RFC, ruptured fibrous cap*.

Prati et al. performed OCT after aspiration thrombectomy and identified 31 PE patients presenting with STEMI. Forty percentage patients with non-obstructive lesions were treated with anti-platelet strategy alone, and the remaining 60% underwent angioplasty and stenting. At a median follow-up of 753 days, all patients were asymptomatic, regardless of stent implantation. They believe that if intra-coronary imaging can verify intact fibrous cap or PE with non-obstructive lesions accurately, the safety and efficacy of these alternative approaches could be considered, consistent with the EROSION study (73). Souteyrand et al. conducted a two-step strategy of invasive management without stenting in ACS patients, guided by OCT. Forty six patients were treated with antithrombotic therapy and performed delayed angiography and OCT in a median period of 6 (3–10) days. PE was detected in 54.3% of patients, PR in 39.1% and calcified nodule in 6.5%. Twenty-three patients, who had an increase in minimal lumen area, benefited from systematic delayed OCT over a median period of 171 days. They believe that it might be a reliable option to choose conservative treatment without stenting in a selected population (PE or non-vulnerable PR without significant stenosis) (74). It might be a reliable option to choose conservative treatment without stenting in a selected population.

In contrary, a prospective multicenter study illustrated that STEMI patients who underwent primary PCI and received everolimus-eluting stent (EES) were divided into in intact fibrous cap (IFC) and ruptured fibrous cap (RFC) groups. Saia et al. showed that IFC and RFC exhibited similar degrees of stenosis and a high incidence of residual endoluminal thrombus after manual thrombectomy, and that the vascular response to the EES was excellent and similar in the two groups (76). Roule et al. compared the characteristics of plaques and thrombi between PR and PE in STEMI patients successfully treated with fibrinolysis by intracoronary optical frequency domain imaging. PE had a lower thrombus volume and burden compared to PR at baseline, while there was no significant difference in thrombotic volume, burden, and its distribution, as well as angiographic estimators of myocardial reperfusion between the two groups after stenting (81). Of note, 52 patients in EROSION study finished 4-year follow-up. All patients were free from hard endpoints while 11 patients underwent elective target lesion revascularization (TLR) due to less improvement in diameter stenosis (82). Therefore, whether plaque morphology underlying STEMI should lead to a different therapeutic approach remains speculative. Future studies are needed to validate this therapeutic approach.

PE compared with PR had a quite different pathological characteristics that may lead to different outcomes. Hu et al. retrospectively evaluated 141 patients with ACS who underwent OCT of the culprit lesion prior to stent implantation. Compared with PR, patients with PE had a lower incidence of distal embolization, higher baseline thrombolysis in myocardial infarction (TIMI) flow grade, and lower incidence of TIMI flow grade ≤2 after stenting. Besides, the rate of revascularization in patients with PE was numerically lower than that in patients with PR, although no statistical difference was found. OCT analysis revealed a higher incidence of malapposition, thrombus, and protrusion in the rupture group compared to the erosion group (75).

Previous studies have shown that patients with PE have fewer lipid plaques, thicker fibrous caps, smaller lipid arcs, and shorter lipid lengths than those with PR (1, 4, 83). Besides, ruptured plaques release highly thrombogenic substrates, inducing recurrent local thrombosis and distal embolization. These findings suggest that PE maybe associated with more favorable outcomes. Higuma et al. compared the post-procedure outcomes of PR and PE. The study revealed that PE had a greater plaque eccentricity index and a lower incidence of the no-reflow phenomenon after PCI than PR. In addition, PE was associated with less microvascular damage after PCI (77). Jia et al. reported that STEMI patients with PE were associated with better myocardial perfusion than those with PR (9). Recently, Niccoli et al. evaluated the prognostic value of PR and IFC in patients with ACS. Compared to patients with IFC, major adverse cardiac events occurred more frequently in patients with PR. Patients with PR as culprit lesions by OCT have a worse prognosis after a 3-year follow-up compared to those with IFC (78). However, White et al. speculated that a higher frequency of the residual incidence of myocardial infarction and stroke was the result of erosion (84). These opposite findings reveal that more clinical trials were needed to explore the association between clinical outcome and criminal plaque morphology.

Hayashi et al. studied 107 patients with acute myocardial infarction (PR: 44; PE: 28; unclassified: 35) using coronary angioscopy and intravascular ultrasound immediately before PCI. Patients with eroded plaques had more pre-infarction angina, less STEMI, lower peak creatine kinase level, less distal embolization after PCI, and less Q-wave MI 1 month after the onset of MI than those with ruptured plaques. Patients with eroded plaque lesions had smaller infarctions than those with ruptured plaque lesions, indicating that eroded lesions were less thrombogenic than ruptured lesions (85). A 3-vessel OCT study was performed to investigate the non-culprit plaque phenotype in patients with ACS based on culprit lesion pathology (PE: 17; PR: 34). None of the PE patients had non-culprit PR, whereas 26% of PR patients had non-culprit PR. The culprit PE group had a smaller number of non-culprit plaques per patient and a lower prevalence of PR, macrophage accumulation, micro-vessels, and spotty calcium in the non-culprit lesions compared with the culprit PR group, which confirmed that PR and PE had distinct pathophysiological mechanisms (3, 25). Several reports suggest more widespread inflammation and instability in culprit PR patients vs. PE patients (5, 66, 86). Sugiyama et al. suggested that PE might involve local endothelial damage rather than widespread coronary arterial inflammation, leading to ACS (87). These results help us to better understand the phenomenon that patients with PE are characteristic with better clinical outcomes compared with PR.

## Conclusion

PE is a common and important pathological mechanism of ACS, and its' pathological features and mechanisms are quite different from those of PR. The mechanism of eroded plaque is still unclear and requires further study. PE is a predictable clinical entity distinct from PR in STEMI patients. Antithrombotic therapy alone maybe safe and effective for some patients with eroded plaques, but needs more clinical trials to support this concept. The long-term prognosis of patients with eroded plaques might be better than that of patients with ruptured plaque.

## Author Contributions

HJ and BY designed the framework and direction of the manuscript. XL, YL, and XBai wrote the manuscript. JQ and XW assisted in the linguistic modification. All authors approved the final manuscript.

## Funding

This research was supported by the National Natural Science Foundation of China (No. 82061130223 and No. 82072031) and Fund of Key Laboratory of Myocardial Ischemia, Ministry of Education (KF202020/LX, KF202019/LY and KF201904/LSY).

## Conflict of Interest

The authors declare that the research was conducted in the absence of any commercial or financial relationships that could be construed as a potential conflict of interest.

## Publisher's Note

All claims expressed in this article are solely those of the authors and do not necessarily represent those of their affiliated organizations, or those of the publisher, the editors and the reviewers. Any product that may be evaluated in this article, or claim that may be made by its manufacturer, is not guaranteed or endorsed by the publisher.
